# 10 Years of Extreme Microbiology: An Interim Reflection and Future Prospects

**DOI:** 10.3389/fmicb.2020.00131

**Published:** 2020-02-05

**Authors:** Andreas Teske

**Affiliations:** Department of Marine Sciences, University of North Carolina at Chapel Hill, Chapel Hill, NC, United States

**Keywords:** extreme microbiology, microbial habitats, microbial physiology, extreme environments, microbial evolution

Gazing into the crystal ball and predicting the future for the next 10 years for Extreme Microbiology can lead to equal shares of amusement and bewilderment down the road, best encapsulated in the exclamation “What were we thinking?” Keeping this caveat in mind while not worrying not too much about it will help coming up with a useful idea or two. Also, looking into what the Extreme Microbiology Section has become should be helpful; reality may contradict even the most elaborate prognosticating efforts, and point into new directions that even the very wise may not foresee.

After surveying the Research Topics and most successful articles of the last 10 years, the first impression is that of a surprising diversity of themes, organisms, contributors, and, by implication, readers. The original “grand challenge article” (Teske, 2010) had certainly invited and welcomed this diversity, but could not foresee its encyclopedic scope. One thing is sure, “Extreme Microbiology” is not the enclave of a handful of specialists who cultivate a strange garden of extremophiles, or craft the Fabergé eggs of microbiology. Many research themes have turned out to be surprisingly popular, notably so in areas where extremophilic microbiology and applicability overlap. Looking through the Research Topics of the last 10 years, one could name investigations into acidophilic sulfur-or iron-oxidizing microorganisms and their importance in mining and bioleaching; the study of thermostable or cold-active enzymes with biotechnological potential; articles on facultative extremophiles and their uses; repeated focus on Actinobacteria, the hardy gram-positive, often thermophilic or thermotolerant bacteria that pervade the terrestrial and soil biosphere; the entire arc of halophile research from natural biodiversity to organism- or enzyme-specific studies; or astro-microbiology under different regimes of scientific gravity, from space station experiments that are actually being done to speculative scenarios of early Martian life. Purists may call this wide thematic range “eclectic” but it is evidence of the vitality of extremophile research that is proceeding in surprising directions. More accurately, they may be “surprising” primarily for senior editors but not so much for the scientists and their audiences who are driving these research efforts.

Some areas of extremophile research integrate the habitats and their microbial inhabitants that reflect its special characteristics; the extreme microbial ecosystem always appears in the background, or shines through. Major habitat types such as hydrothermal vents, hydrocarbon seeps or the deep subsurface, provide the umbrella under which the respective microorganisms or microbial communities make their appearance. Extreme habitats require well-matched extreme microbial inhabitants, distinguished by uniquely adapted biochemistry and intriguing physiological capabilities that reflect the conditions of their existence. For many authors and readers [including this editor], this flavor of extreme microbiology—ultimately, extreme microbial ecosystem research—has drawn them into this field. For example, who would not be amazed by the complex biogeochemical layering and microscale architecture of a microbial mat from a hot spring or hydrothermal vent that may have existed in similar form already billions of years ago? Views and downloads for some articles of this type exceed 5,000 and 500, respectively, showing their enduring interest.

However, it would be short-sighted to think that visually charismatic microbial ecosystems and expedition targets will necessarily define the attraction of Extreme Microbiology in the coming decade. Planet Earth, and perhaps not only planet Earth, will continue to be searched for extreme microbiomes, but this is not where the story ends. Now we come to the risky part of this short editorial, prognosticating. Be it! I think that the most exciting scientific advances of the next decade in the broadly defined “extremophile” area will target the uncharted depths of microbial evolution. I suggest (1) microbial dark matter and (2) archaea as key areas to watch and to cultivate in Frontiers, and I am very pleased to see that a brand-new specialty within Frontiers in Microbiology, “Biology of Archaea,” will now provide a permanent home for these new research developments. “Microbial dark matter” is shorthand for the vast diversity of uncultured microbial life that is out there (Rinke et al., 2013), and currently eludes laboratory cultivation or other forms of domestication. The outlines of the bacterial domain have changed dramatically, from a tree into a forest; and just as in a real forest, many lineages survive and thrive only by some form of symbiotic or parasitic association (Hug et al., 2016). The archaeal domain has changed from a collection of interesting specialties into a vast evolutionary frontier where all of a sudden everything seems possible. For example, hydrocarbon metabolism has proliferated throughout the archaeal domain in the last 2 or 3 years (Seitz et al., 2019); the proposal that archaeal lineages—the Asgard archaea—provided genetic and structural machinery for the ancient eukaryotic host cell has fired up the field of microbial evolution; the archaeal tree of life is at last emerging in full and draws attention to the biogeochemical and ecological roles of the archaeal biome (Spang et al., 2017). Elucidating microbial dark matter and charting the archaeal world will engage a new generation of investigators and introduce them to the Frontiers community. As chief specialty editor, I will launch a Research Topic on archaea in their environments—co-hosted with the “Biology of Archaea” section—as my contribution to celebrate the next 10 years of Frontiers in Microbiology.

This editorial was written at sea on a research ship, the JOIDES *Resolution*, where a multidisciplinary team of geologists, geochemists and microbiologists is exploring microbial life in the layered hydrothermal sediments and basalts of a young oceanic spreading center, Guaymas Basin in the Gulf of California [joidesresolution.org/expedition/385/]. Guaymas Basin is famous for its microbial diversity hosted by its hydrothermal vents, hydrocarbon seeps and organic-rich seafloor sediments (Figure 1) (Teske et al., 2014, 2016). The ship is a floating university with well-equipped labs for every research direction and a major drill rig riding midships, to recover sediment and basalt from the hydrothermal zone several hundred meters below the seafloor. Here, the wider geothermal and geochemical properties of this extreme subsurface environment, and the types of microbial life to be tracked are constantly evaluated in relation to each other. Finally, the microbiomes of this special place will illuminate the uncharted depths of microbial evolution.

**Figure 1:**
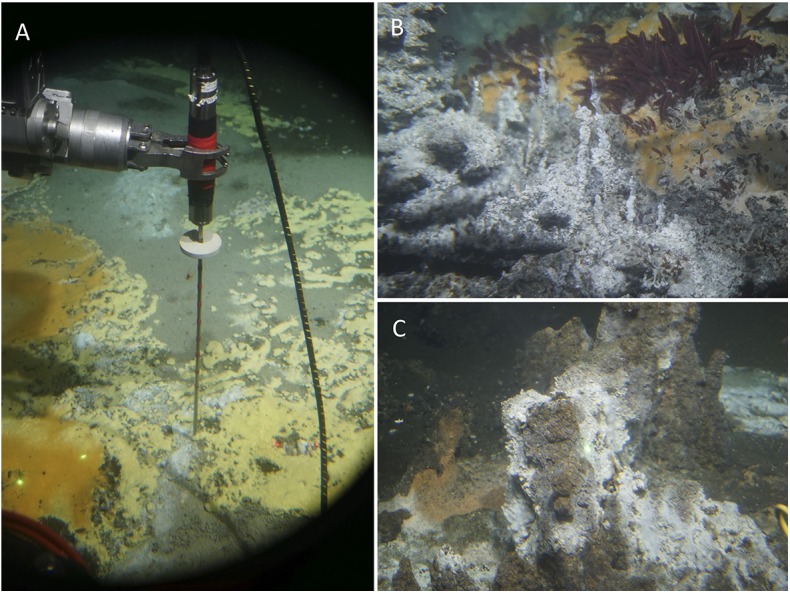
Extreme seafloor habitats of Guaymas Basin. **(A)** Sulfidic, methane-saturated seafloor sediments with strong hydrothermal temperature gradients, to be measured with the heatflow probe of submersible *Alvin*. Sediments such as these host great microbial diversity within their steep thermal and redox gradients (Dombrowski et al., 2018). **(B)** Miniature chimneys precipitated from ca. 100°C hot hydrothermal fluids, surrounded by shimmering warm water. **(C)** Small mound consisting of red-brown petroleum-stained sediment concretions, lightly covered with sulfur precipitates. Photos from Expedition AT42-05 in southern Guaymas Basin, Nov. 15 to 29, 2018. Courtesy of WHOI/Alvin group.

However, one example can go only so far. Authors who publish in Frontiers in Microbiology, and the Extreme Microbiology section, will continue to launch diverse investigations where physiological and genomic detail can ultimately be linked to organisms, communities, and entire ecosystems. In the future, I hope that the readers of Frontiers in Microbiology will find more examples of these exciting endeavors in the “Extreme Microbiology” section.

## Author Contributions

The author confirms being the sole contributor of this work and has approved it for publication.

### Conflict of Interest

The author declares that the research was conducted in the absence of any commercial or financial relationships that could be construed as a potential conflict of interest.
